# Activated carbon from biomass precursors using phosphoric acid: A review

**DOI:** 10.1016/j.heliyon.2022.e11940

**Published:** 2022-12-01

**Authors:** Ibsa Neme, Girma Gonfa, Chandran Masi

**Affiliations:** a Department of Chemical Engineering, College of Biological and Chemical Engineering, Addis Ababa Science and Technology University, Addis Ababa, Ethiopia; b Department of Biotechnology, College of Biological and Chemical Engineering, Addis Ababa Science and Technology University, Addis Ababa, Ethiopia; c Biotechnology and Bioprocess Center of Excellence, Addis Ababa Science and Technology University, Post Box: 16417 Addis Ababa, Ethiopia; d Nanotechnology Center of Excellence, Addis Ababa Science and Technology University, Post Box: 16417 Addis Ababa, Ethiopia

**Keywords:** Lignocellulosic biomass, Activated carbon, Phosphoric acid, Chemical activation, Parameter

## Abstract

Low-cost and renewable adsorbent activated carbon from lignocellulosic biomass is a focus of worldwide concern due to its readily available waste disposal problems in the environment. Physical and chemical processes are the main procedures forproduction of biomass-activated carbon (AC). Activating lignocellulosic biomass by chemical methods in terms of energy performance, lower timecarbonization, and temperature is mutual forthe production of activated carbon. Out of chemical activating agents (H_3_PO_4_, H_2_SO_4_, ZnCl_2_, FeCl_3_, NaOH, KOH, and K_2_CO_3_), H_3_PO_4_ is the most commonly used chemical activating agent during the synthesis of activated carbon from lignocellulosic biomass because of its ease of recovery, low environmental impact, and higher carbon yield. The surface area of most activated carbon from lignocellulosic biomass by H_3_PO_4_was in the variability of 456.1–2806 m^2^/g, yielding 26.1–85 % and an extreme adsorption capacity of 2.5–89.29 mg/g. And also, high acids to precursor ratio and activation temperature of AC were synthesized from lignocellulosic biomass. Generally, the advantage of this review paper, gathers evidence from currently published articles deliberating chemical composition, proximate values, biomass activation methods, the elemental composition of lignocellulosic biomass, physio-chemical properties of different lignocellulosic materials AC synthesized using a phosphoric acid activation agent, and the usage of derived activated carbon through phosphoric acid activation for water disinfection, solute organic matter, energy storage, and heavy metal removal.

## 1 Introduction

Activated carbon is a carbonous solid widely used as a multipurpose adsorbent meant for the adsorption of liquid and gaseous phases. Activated carbon (AC) is produced from anextensive range of precursors, including char and agricultural waste [1]. Surface chemistry, microporosity, and porosity all affect how well AC can be removed from gas or liquid phases [2]. Activated carbons are made by choosing the right raw materials and making sure they cost many billions of dollars per year [3].

Generally, activated carbons are classified as commercial and low-cost types. Most commercial activated carbons are made from materials that can't be reused, like coal [4], animal bones [5], and apricot stone [6]. Low-cost activated carbons are made from renewable biomass or agricultural waste, like a lotus stalk [7], sewage sludge [8], waste tires [9], coconut shell [10], cane bagasse [11], almond shell [2], acorn shell [12], peanut shell [13], palm kernel shell [14], corncob [15], and Canarium Schwerin fruit Nutshell [16]. Commercial activated carbon is more expensive than non-commercial activated carbon because it is produced from non-renewable raw materials [17]. As a result, the majority of the numerous research efforts have focused on low-cost and environmentally friendly substitute constituents that can be converted into low-cost activated carbons, and the carbon content of these biomass precursors is lower when compared to non-renewable precursors like coal, anthracite, or peat. Nonetheless, their availability, low cost, and non-harmful nature have a greater impact than their lower carbon content and yields [18, 19]. The numerous investigations have been conducted about the synthesis of AC from various lignocellulosic materials through chemical activation of H_3_PO_4_ [20], H_2_SO_4_ [21], ZnCl_2_ [22], FeCl_3_ [23], NaOH [24], KOH [25], K_2_CO_3_ [26], CO_2_ [27], steam [28], composite ZnCl_2_–CuCl_2_ [29], H_2_O–CO_2_ [12] and physical methods (activation by steam, and carbon dioxide) [30], biological activated carbon (BAC) [31, 32], and hydrothermal carbonization [33, 34]. When compared to physical, biological, and hydrothermal activation with chemicalactivation, chemical activation methods are the most favoredas a result of low energy price, lower time of pyrolysis, high surface area, and more activated carbon yield products. Out of the chemical activation agents, phosphoric acid is further chosen due to its excellent synthesis of mesopores, resulting in higher total pore volume and diameters [18]. It delivers a familiar method to recover the AC product throughout the processing step which is only washing with water is required [35]. And also, when compared to ZnCl_2_, H_3_PO_4_ is the furthermost favoredas a result of the eco-friendly drawbacks related to ZnCl_2_ [36,37]. Additionally, AC synthesized with zinc chloride cannot be applied in food and medicinal factories as they may infect the product [18]. Up until recently, no review has been conducted on the many articles on the research conducted by numerous scholars on the synthesis of activated carbon (AC) from lignocellulosic-based activation by phosphoric acid and its performance in the elimination of volatile matter [38]and yields of activated carbon. This review work is therefore intended to provide adequate knowledge of the efforts made by innumerable investigators concerning the synthesis of AC from lignocellulosic materials activated by phosphoric acid and its advantages over other activation chemicals [11].

### 1.1 Precursor for synthesis of AC

**Figure 1:**
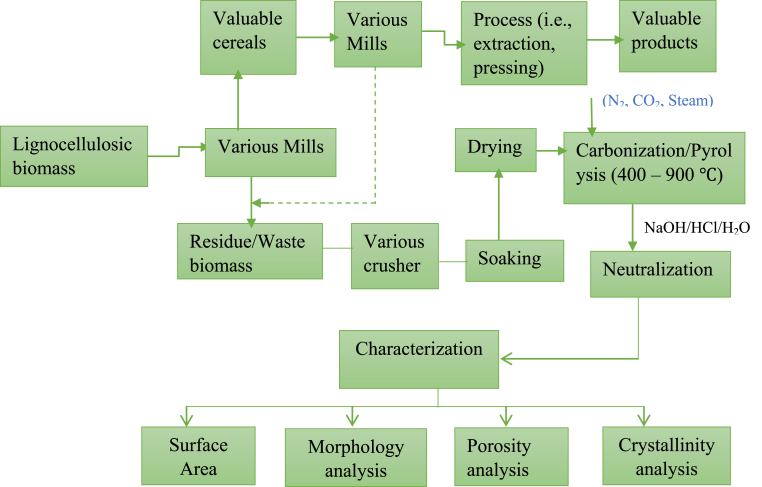
Mutual process flow diagram of lignocellulosic biomass conversion to activated carbon.

Activated carbon can be synthesized from renewable or non-renewable carbonaceous precursors. It has been classified based on its initial precursor [19]. The kinds of precursor, impurities, activating agents, activation time, temperature, and acid-to-precursor ratio show a vital character in influencing the excellence, yield, and characteristics of the resulting AC [38]. The precursor selected for AC synthesis is principally based on availability, cost, and constancy of supply [27]. The use of any lignocellulose precursor for the production of AC has been observed by numerous investigators [18], and also any agricultural residue which has high or low organic content can be used for activated carbon production [39]. Figure 1 explains the diagram of biomass conversion to activated carbon using chemical activation methods.

### 1.2 Chemical composition of the lignocellulosic biomass

**Figure 2:**
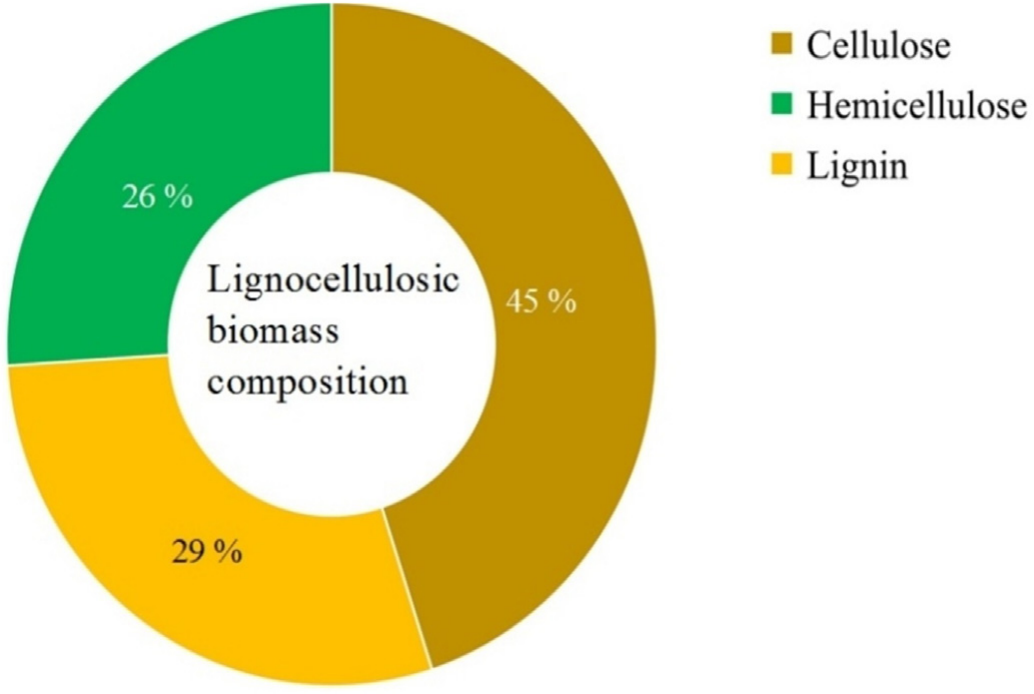
General (average) chemical composition of lignocellulosic biomass.

Carbon content biomass is made of fiber (cellulose) and has the right properties, like activation time, temperature, and the impregnation ratio [19]. Because of activated carbons' multipurpose nature, their global market has recentlyhemicellulose and lignin. Cellulose is the most abundant mass component of the most common types [40]. Hemicellulose is a polysaccharide containing short divisional chains of sugar. It is typically composed of the pentoses D-xylose and L-arabinose and the hexoses D-glucose, D-mannose, and D-galactose, with minor quantities of L-rhamnose [41]. Lignin has a complicated molecular structure containing cross-linked phenolic polymers [42]. Lignocellulose biomass chemical composition differs by variety and production location and also changes significantly during biomass processing [43]. Biomass can be transformed into adsorbents, biofuels, and bioenergy in different ways. In the case of biomass conversion to an adsorbent or activated carbon, the chemical composition of each is a key characteristic to know before the synthesis of any AC. Table 1 displays the composition of some lignocellulose biomass and their proximate studies, namely, fixed carbon contents (FC), volatile matter [44], ash contents (A), and moisture (M) for precursor and after the conversation to activated carbon. Figure 2 shows the general (average) composition of lignocellulosic biomass.

**Table 1:** Chemical composition and proximate values of some lignocellulosic biomass.

Lignocellulosic biomass	Proximate analysis value				Chemical composition			References
	Moisture contents (%)	Ash contents (%)	Volatile matter (%)	Fixed carbon (%)	Cellulose (%)	Hemicellulose (%)	Lignin (%)	
**Tomato leaves**	2.68	25.72	77.35	10.98	10.91	8.13	24.86	[45, 46]
**Barley straw**	11.5	5.3	76.2	18.5	56.2	7	9.2	[47]
**Jatropha shell**	1.02	6.81	82.43	9.74	34	40	12.7	[48]
**Coconut shell**	10.1	3.2	75.5	11.2	17.89	56.29	25.82	[49]
**Durian shell**	2.95	5.67	72.56	18.82	12.46	33.2	17.93	[50]
**Almond shell**	6.55	6.85	75.1	17.1	21.72	27.74	36.12	[2, 51]
**Sunflower shell**	Nil	1.8	81	17.2	67.1	Nil	27.1	[52]
**Karanja fruit hull**	Nil	1.54	82.37	14.06	33.72	25.18	38.62	[53]
**Benth fruit shell**	2.7	4.7	78.2	17.1	45.4	6.4	30.1	[48]
**Coffee husk**	Nil	7.17	74.71	18.12	30	22.5	17.4	[51]
***Delonix regia* fruit pods**	0.22	2.8	92.03	Nil	13.9	24.13	23.36	[54]
**Walnut shell**	6.8	1.3	55.3	35.3	27.9	30.2	39.1	[55]
**Soya husks**	6.3	5.1	69.6	19	36.43	12.5	18.2	[56]
**Corn Kernel**	0	2.3	79.03	54.28	42.2	1.9	38.7	[57, 58]
**Banana empty fruitbunch**	5.21	15.73	78.83	Nil	8.3	21.23	19.06	[54]

#### 1.2.1 Cellulose

Cellulose is any carbon-based matter that is available in renewable biomass like agricultural harvests, soft and hardwoods, grasses and flowers, municipal wastes, and other residue materials [56]. Cellulose is the most plentiful carbohydrate polymer and acts as a structural apparatus for plant cells with lignin. Its biomass composition ranges between 35 and 50%. In recent times, cellulose is mostly used for the production of bioethanol [56].

#### 1.2.2 Hemicellulose

Hemicellulose, a natural polymer found in lignocellulosic biomass, follows cellulose in abundance [57]. The component of hemicellulose in biomass is 20–35%. Arabinose, mannose, galactose, glucose, and xylose are the most common monomers that occur in hemicellulose. Hemicellulose is more hygroscopic, open-structured, and non-crystalline than cellulose [58].

#### 1.2.3 Lignin

Lignin is also the furthermostplentiful biopolymer lignocellulosic biomass, and its component is (20–30%) [59]. The structure of lignin changes throughout cell development and its composition depends on environmental conditions. The arrangement of lignin in many lignocellulosic biomasses is quite different [60].

## 2 Proximate analysis of lignocellulose biomass

The proximate examination is the quantification of moisture contents, ash contents, volatile matter [44], and fixed carbon (FC) and has been broadly used for more than 160 years [61]. It provides an extensive understanding of biomass burning properties and estimates the emission of constituents like sulfur [62].

### 2.1 Moisture contents

The quantity of water existing in lignocellulosic raw materials and quantified as a proportion of the whole precursor mass is called moisture content. It is dependent on the kind of biomass, ranging from 15% in biomass straw to 90% in algae biomass [63]. In storage, degradation of biomass is strongly associated with moisture content and allows for safe long-term storage of lignocellulosic biomass [64]. Commonly, the higher the moisture content of biomass, the more energy is required for size reduction [65].

### 2.2 Ash contents

Ash is the residue obtained after the burning of coal or biomass. Based on the combustion of coal or lignocellulosic biomass, there are two types of ash residue generated [66, 67]. The first one is biomass bottom ash, synthesized in the incineration chamber that is composed of sands, mostly vitreous silica [44]. The second one is fly ash, which is the filtrate separated from gases outside the incineration chamber and it contains a fraction of inorganic or organic unburned carbon [68]. The properties of the biomass used, incineration technology, and operating conditions of the burning are the keyinfluences that affect the quality and quantity of biomass ash in the burning of lignocellulosic biomass [69]. When wood biomass is compared with agricultural residue, the biomass of wood has lower ash content than agricultural residue [66]. Agricultural biomass ash consists of different metal impurities like silicon, calcium, magnesium, aluminium, potassium, sodium, and titanium. All of these metal contents are commonly used as deacidifying soil, in the production of ceramics, andin the production of slag [70, 71].

### 2.3 Volatile matter

The percentage loss when coal or biomass is heated without air for moisture content adjustment is called volatile matter [61]. It usually contains a combination of long and short chains of hydrocarbons, aromatics, some sulfur, and carbon dioxide gases. Naturally, for many kinds of lignocellulosic biomass, the volatile matter is high. The quantity of volatile matter extant is strongly reliant on the nature of the carbonized biomass and the pyrolysis situations, activation temperature, and heating rate [72].

### 2.4 Fixed carbon cellulose

Fixed carbon (FC) is the compact carbon in the lignocellulosic biomass and coal, which remains in the ash during the incineration process after reactivation. It depends on the amount of volatile matter and is calculated with the present subtraction of moisture, ash, and volatile matter from one another [61]. Differences in heating rates will have a big impact on both the amount of ash and the amount of volatile matter that is made, as well as the amount of "fixed carbon" that is calculated [73].

## 3 Method of biomass activation for the synthesis of AC

As a whole, activated carbonis prepared from various biomasses using physical activation, chemical activation, biological activation [31, 32], and hydrothermal carbonization processes [74, 75]. The physical activation method comprises the conversion of precursors to bio-char [fixed carbon mass] using inert gases like steam, carbon dioxide, or gas mixtures during pyrolysis [76]. Meanwhile, chemical activation methods such as nitric acid (HNO_3_), phosphoric acid (H_3_PO_4_), sulfuric acid (H_2_SO_4_), and alkali (e.g., NaOH, KOH), or salts such as zinc chloride [77] have been used. In terms of energy efficiency, hydrothermal carbonization (HTC) is an efficient technique for the synthesis of activated carbon compared to physical and chemical activation processes [78]. The physical activation causes difficulties, and technical problems, particularly when it is used with steam organized by the inert gas, and lastly, the advanced temperature is regularly practiced throughout the activation process [79]. The chemical activation process requires a lower time of pyrolysis and a lower activation temperature [76]. The Biological activated carbon (BAC) methodpropositionsnumerousadvantages to known watertreatment proceduresandthis is an actual and cost-inexpensive means for eliminating biodegradable organic matter through the microbe's takeoverof the activated carbon exterior [80]. However, biological activated carbon treatment techniques cannot biologically eliminate organic carbon beyond biodegradable dissolved organic carbon and filters become seemingly unproductive subsequently around a month of a continuous process [32, 81]. Hydrothermal carbonization does not use custom organic solvents, surfactants, or catalysts; hence it is called green methods [61] and HTC is more powerfully positive. It does not need pre-drying of the precursor [82]. However, there is a rare weakness to be considered, for instance, the consumption of water in a high quantity and the fluid effluent handling that rise the waste cost [83]. Hence, activation of carbon content precursors with H_3_PO_4_ has become an ever more popular technique for the huge-scale synthesis of ACs since the use of this chemical has some eco-friendly benefits, for example, ease of repossession, low energy price, and more activated carbon products in most recent studies [59].

### 3.1 Chemical impregnations

Several chemical impregnation techniques have been used in previous works to improve the exterior chemistry of activated carbons (ACS). Amongst these techniques, conservative solution impregnation is one of the most commonly used [84, 85]. The surface interaction of the AC can be proficiently improved through chemical impregnation that, organized by the inherent nature of the AC, can extremely increase the adsorption volume [86]. Most research studies [23] have previously reported the development of adsorptive characteristics of AC with strong acids such as H_3_PO_4_, H_2_SO_4_, alkali (i.e., NaOH, KOH), and metal species salts (i.e., Zn, Cu, Fe). Out of those activating chemicals stated, the most significant and normally applied to activate chemicals are phosphoric acid, zinc chloride, and alkaline metal compounds. H_3_PO_4_ and ZnCl_2_ are applied for the treatment of carbon-containing resources that have not been carbonized earlier [87]. But, rather than ZnCl_2_, H_3_PO_4_ is preferable because of environmental difficulties related to ZnCl_2,_ [59]. Additional AC synthesized using phosphoric acid has good acceptance in nutrition, water, as well as chemical and pharmacological needs because of its non-contaminating nature [87].

#### 3.1.1 Phosphoric acid as activating agents for biomass

**Figure 3:**
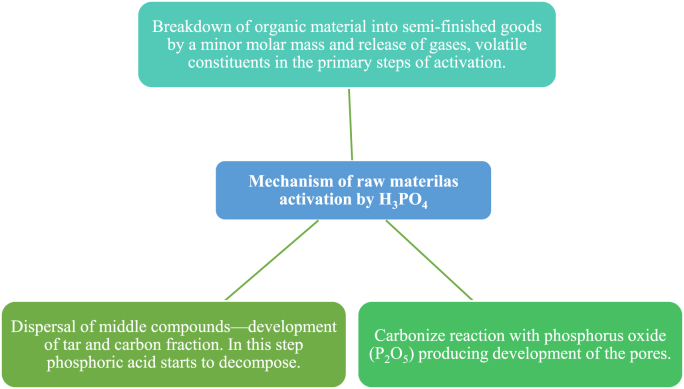
Mechanism of the biomass activation steps utilizing phosphoric acid.

Chemical activation encompasses pyrolysis and activation in a sole step in which the fresh biomass, soaked in convinced chemical agents, is thermally decomposed. The most common activating chemicals are H_3_PO_4_, ZnCl_2_, H_2_SO_4_, sodium hydroxide, potassium hydroxide, and others [88]. Phosphoric acid shows vital activity in the bond split and the removal of the bio-polymers/water at low temperatures and is used as an activating acid agent in the activated carbon synthesis [89, 90]. As reported in previous studies, methods of H_3_PO_4_ biomass treatment start with cellulose depolymerization, followed by biopolymer dehydration, the creation of aromatic rings, and lastly, the removal of phosphate cluster [88]. A weak acid may also be valuable to hydrolyze cellulose to cellulase enzymes and it can live with the existence of salts possibly resulting from agricultural biomass, however strong acids willingly cause cation conversation, like proton leaching [91]. The extra addition of water to concentrated phosphoric acid causes hydrolysis of cellulose to occur through an esterification reaction [92]. And according to the previous studies [93], the mechanism of the raw material activation utilizing phosphoric acid can occur in three steps indicated in Figure 3.

According to Yaxin Li et al. [94], phosphoric acid might also syndicateby organic material in biowastes to make phosphate and polyphosphate bonds that attach and cross-linkage polymer fragments.

The activation mechanism and the variations that the acid undertakes in the specific temperature ranges are described from Eqs. (1), (2), (3), (4), (5), and (6).

At a temperature range of 100–400 °C

(1) 2H_3_PO_4_ → H_4_P_2_O_7_ + H_2_O

(2) 3H_3_PO_4_ → H_5_P_3_O_10_ + 2H_2_O

(3) nH_3_PO_4_ →H_n+2_P_n_O_3n+1_ + (n – 1) H_2_O

The adsorbed water is released and phosphoric acid is arid. As an outcome of the breakdown of the functional set CO_2_ and CO are released.

At a temperature range of 400–700 °C

(4) H_n+2_P_n_O_3n+1_ → P_4_O_10_ + H_2_O

(5) P_4_O_10_ + 2C → P_4_O_6_ + 2CO_2_

Due to dehydration of phosphoric acid (H_3_PO_4_), water (H_2_O) is released, and the resulting Di-phosphorus Penta oxide (P_4_O_10_), as a sturdy oxidant, reacts by carbon to make new and widening present pores, releasing carbon dioxide and as a result of the surface functional groups breakdown carbon monoxide is formed.

At temperatures 700–800 °C

(6) P_4_O_10_ or P_4_O_6_ + CHx → PH_3_ + CO_2_ or CO

From Eq. 6, PH_3_ is created, and CO_2_ and CO are out as a consequence of reactions and the breakdown of functional groups. All these steps happen in the biomass pyrolysis process [94].

The previous information regarding the oxidation safety provided via phosphorous composites presented that the phosphorus compounds can evaporate from the carbon surface at temperatures higher than 800 °C and the reaction products permit the surface to create and produce pores [95].

### 3.2 Ultimate analysis of activated carbon

The ultimate examination is used to characterize biomass composition in wt. % carbon, hydrogen, nitrogen, oxygen, and sulfur (if any). It also delivers a suitable technique for reporting the main organic elemental constituents of lignocellulosic biomass [96]. The carbon and hydrogen content in biomass increases the heating value, while the oxygen content decreases the heating value of biomass during pyrolysis. Nitrogen and sulfur content are the sources of NOx and SOx, respectively [97]. The ultimate analysis of lignocellulosic precursors is organized in Table 2.

**Table 2:** Elemental composition of lignocellulose raw materials precursors.

Precursor	Elemental composition					References
	C^a^ (wt.%)	H^b^ (wt.%)	N^c^ (wt.%)	O^d^ (wt.%)	S^e^ (wt.%)	
**Wheat straw**	52.3	9.4	1.9	35.8	0.6	[98, 99]
**Bagasse**	41.55	5.55	0.03	52.86	Nil	[100]
**Corn stover**	47	5.66	0.65	41.4	0.06	[101]
**Chinese fir sawdust**	48.95	6.54	0.11	39.2	Nil	[102]
**Saw dust**	45.34	6.02	0.53	47.05	1.07	[103]
**Timothy grass**	49.2	9.3	2.2	38.4	0.9	[98, 99]
**Switchgrass**	47.3	5.31	0.51	41.6	0.1	[101]
**Rice stalk**	40.79	7.66	1.17	49.89	0.49	[104]
**Pine wood**	54.4	9.6	1.6	34	0.4	[98, 99]
**Bamboo**	45.53	4.61	0.22	Nil	Nil	[105]
**Rice husk**	35.6	4.5	0.19	59.7	0.02	[106]
**Olive stone**	44.8	6	0.1	49.09	0.01	[107]
**Palm kernel shell**	48.48	6.06	0.79	44.67	0.35	[108]
**Acacia cincinnata biomass**	48.47	5.5	0.82	45.21	Nil	[109]
**Loblolly pine chips**	50.5	6.26	0.09	42.6	Nil	[110]
**Napier grass stem**	51.61	6.01	0.99	41.07	0.32	[111]
**Typha angustifolia**	52.895	5.844	1.217	40.044	Nil	[112]
**Castor seed**	29.288	3.914	Nil	29.84	0.03	[113]
**Sweet sorghum**	22.08	6.52	0.21	71.2	Nil	[114]
**Durian shell**	60.31	8.47	3.06	28.06	0.1	[115]
**Sago biomass**	39.66	6.61	0.19	53.54	0.00	[111]
**Peanut shell**	50.64	6.86	1.18	41.32	Nil	[116]
**Palm shell**	19.74	5.32	0.08	44.86	0.16	[117]
**Elephant grass**	44.5	5.4	1.4	31.8	Nil	[118]
**Bagasse jatropha**	40.94	7.42	0.26	51.14	0.24	[118]
**Sunflower biomass**	35.83	5.2	0.16	58.55	0.26	[109]
**Corn cob**	41.16	5.11	0.46	53.27	0	[119]
**Rice husk**	30.4	4	0.9	21.1	Nil	[117]

a Carbon

b Hydrogen

c Nitrogen

d Oxygen

e Sulfur

### 3.3 Effects of basic factors on yield, surface area, and textural of activated carbon

During the synthesis of activated carbon acid to precursor ratio, activation time and temperature, and sometimes size of precursor are crucial factor parameters on the yield, surface area, and pore volume of activated carbon. The main effects of those factors were discussed one by one below.

#### 3.3.1 Acid to precursor ratio on yield and textural characters of activated carbon

The acid to precursor/impregnation ratio is the ratio of the mass of the chemical agent to the precursor [18, 19]. The structural character of activated carbon is related to raw materials, activation time, temperature, and acid to the precursor ratio of activating agents [120]. During the synthesis of activated carbon (AC) from lignocellulosic materials, as the impregnation ratio increases, the products of activated carbon decrease as a result of the reaction between the activationacid and volatile matter [44] of precursors in the activation procedure, and also the structure, or surface area, of activated carbon increases with impregnation ratio. Similar observations were made by Redzuan [93], Lim [87], and Lee [121]. However, some previous studies, like Olawale [16], indicated that as the impregnation ratio increases, the yield of AC also increases. This is related to the precursor variation, which causes a slight increase in yield, which is worth noting. Acid hydrolysis decomposes the polymers (e.g., cellulose) into slighter molecules, finally, sugars hydrolyze esters or ethers, thus these slighter fragments develop more reactive and tend to decompose with the creation of slighter molecules [93]. At low crosslinking ion gases, liquids and char were formed. Activated carbon at a high impregnation ratio has a high pore volume compared to a low impregnation ratio [122]. In other cases, the quantity of phosphoric used for impregnation was analyzed through the degree of impregnation ratio (IR) calculated by the eq. (7).

(7) $$IR=\frac{m_{p}}{m_{b}}$$

where, mp = mass of phosphoric acid (g) calculated from density of H_3_PO_4_, mb = mass of biomass (g) [123].

**Figure 4:**
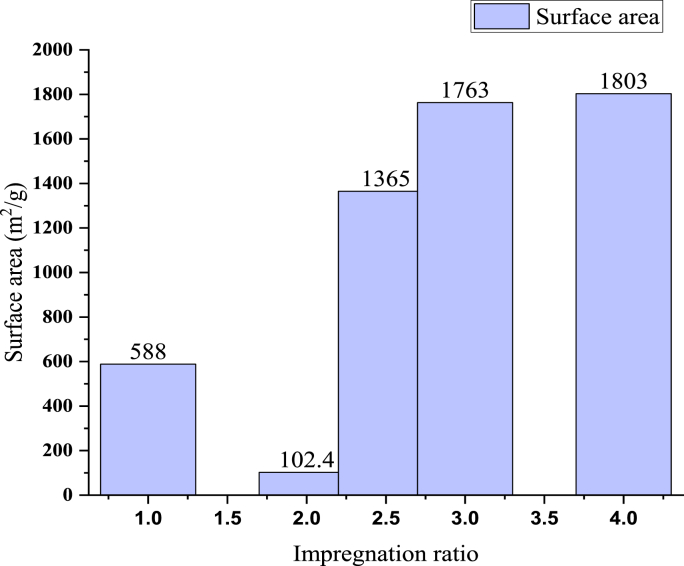
Effect of impregnation ratio on a surface area of activated carbon from rice husk, preparation parameters T = 500 °C, and t = 60 min from [20, 124, 125, 126].

Figure 4 indicated the effects of the impregnation ratio on the surface area of activated carbon synthesized from rice husk via phosphoric acid at a constant 500 °C activation temperature and 60 of activation time respectively.

#### 3.3.2 Effect of activation temperature on yield and textural properties of activated carbon

Activation of biomass at high temperatures is a basic technique of treatment of the sorption parameters of the activated, which creates new functional groups on the surface [127]. Increasing the activation temperature reduces the activated carbon yield due to lignin incineration in biomass and the release of more volatile compounds [128]. Increasing activation temperature also increases narrow micropores and widens the established micropores using activating chemicals due to the melting of compounds found in biomass [129]. Most of the previous studies supported this statement in their research papers. The mutual ranges of activation temperature in the synthesis ofAC by chemical activating agents were 400–900 [93,130,131].

#### 3.3.3 The consequence of activation time on the yield and textural character of AC

Activation time and other parameters that affect the yield and structure of activated carbon also vary. According to previous literature, the yield of activated carbon decreases significantly as activation time increases. However, it is directly proportional to surface area and porosity due to the long reaction between the activation agent and biomass [28, 132]. The micropore of AC will tend to become mesoporous and macropore and the specific surface area of activated carbon will be reduced because of the loss of carbon for a lengthy activation time [133, 134].

#### 3.3.4 Effect of activation time, temperature, and impregnation ratio on the surface morphology of AC

The activated carbon is classically smooth whereas occupied with more pores. This porosity of AC was observed by Scanning Electron Microscope [92]. Before any activation or treatment of biomass, there is no pore on the surface of lignocellulosic biomass. However, after pyrolysis of biomass at various activation times, temperature, and impregnation ratios, the pore/surface morphology of activated carbon developed more. The produced AC has typically a large pore ranging from 20 to 190 μm [135]. The impregnation ratio and activation temperature have been effective in producing well-established pores on the biomass-activated carbon as a result of depolymerization and subsequent release of volatile organic substances from carbonization [134].

### 3.4 Properties and characteristics of phosphorus impregnated AC

Activated carbon (AC) can be synthesized by physical or chemical activation methods. However, chemical activation has extra benefits over physical activation due to maximum carbon yield, high surface area, and well-grown porous texture in carbon [136]. Chemical activation also develops oxygenated surface complexes on the surface of activated carbon. The surface oxygen functional groups can be simply introduced to the carbon through diverse techniques of activation like the reaction between the carbon surface and solutions of oxidizing agents like H_3_PO_4_. Phosphoric acid activation is commonly preferred over zinc chloride agents as a consequence of ZnCl_2_ environmental influence and its activated carbon cannot be used in food, water, and pharmaceutical industries [87]. Chemical activation utilizing H_3_PO_4_ can simply expose pores and voids of the synthesized AC to improve its adsorption capability. Earlier, phosphoric acid activation has been applied to different lignocellulosic biomass such as cashew nut shells [137], palm kernel shells [121], pecan shells [138], durian shells [139], cotton stalk [122], pumpkin seed shell [140], nutshell [16], limonite acidissima shell [141], groundnut shell [142], almond shell [142], and date palm park [143] to produce low-cost activated carbon. Some literature on activation by phosphoric acid and correlated specifics about AC synthesized by this acid are enumerated in Table 3.

**Table 3:** Review of activated carbon parameters and physio-chemical properties from different lignocellulosic materials using phosphoric acid activation agent.

Precursor	Activation parameters			Physio-chemical properties				References
	Temperature (°C)	Activation time (min.)	Impregnation ratio	Surface area (m^2^/g)	Pore volume (cm^3^/g)	Pore diameter (nm)	Yield (%)	
**Kenaf short fibers**	600	30	3:1	1570	0.63	3.96	40	[144]
**Baobab fruit shells**	700	150	1:1.75	1089	0.3464	1.45	80	[145]
**Cashew nut shells**	600	120	1.7:1	1514	1.147	---	85	[137]
**Mustard straw**	768	60	4.2	498	0.32	2.64	Nil	[146]
**Pine wood sawdust**	800	15	1:1	1079	0.53 ± 0.06	Nil	---	[147]
**Palm kernel shell**	600	120	1:1	456.47	0.13	0.63	---	[121]
**Corn cob**	400	120	1:2	700	0.011	---	---	[148]
**Date palm bark**	400	180	40 wt.%	902	0.49	2.16	30	[143]
**Maize tassel**	700	30	1:2	1262.5	1.54	---	---	[149]
**Coconut shell**	600	120	1:1	479.17	0.15	0.62	---	[121]
**Palm shell**	475	75	1.75	2000	1.2	-	45–55	[150]
**Corn straw**	300	120	1	463.89	0.387	Nil	Nil	[151]
**Palm shell**	425	30	3	1109	0.903	3.2	<30	[150]
**Lotus stalks**	450	60	40 wt.%	1418.78	1.253	3.53		[7]
**Kanlow Switchgrass**	900	60	---	1373	1.43	---	---	[152]
**Durian shell**	500	20	30 wt.%	1404	Nil	Nil	Nil	[139]
**Palm shell**	855	135	9.42	615	0.28	--	---	[14]
**Cotton stalk**	500	120	1.5	1720	0.89	Nil	56.8	[122]
**Camellia oleifera shell**	800	60	3:1	1076	1.17	Nil	56	[153]
**Fox nut shell**	700	60	1.5	2636	1.53	2.32	37.84	[59]
**Grape seed**	500	120	1:3	1139	0.24	3.5	50	[154]
**Pumpkin seed shell**	500	60	2	1421	0.908	Nil	31	[140]
**Paulownia wood**	400	60	4	2806	1.746	--	42.6	[155]
**Raffia palm shell**	523.68	103.83	1:4	456.1	0.25	2.13	77.98	[156]
**Soap nutshell**	500	90	1:1	1287.77	0.89	0.139	40.97	[157]

#### 3.4.1 Properties compared toanother chemical-based impregnation

During the impregnation step, monitoring physical or chemical exchanges happening in the bulk solution with the substratum is difficult because of the high polarity of phosphoric acid [158]. This means the concentration of acid solution has a key influence on the activation process with phosphoric acid [18]. Phosphoric acid, rather than other acids, basic and neutral activation [159], is preferred to give extreme yield, surface area, and porosity of activated carbon (AC) due to its growth of microporous and mesoporous pores in AC [140]. Phosphoric acid has two important applications: it recovers precursor incineration decomposition and develops a lattice structure [2]. But, the excessive amount of H_3_PO_4_ owing to the development of a protective coating on AC does not result in an increment of porosity [160]. Table 4 shows the properties and efficiency of phosphoric acid impregnation of activated carbon compared with other chemical agents.

**Table 4:** Evaluation of the useful properties and textural features of AC synthesized from numerous biomass and activation with phosphoric acid.

Precursor	Impregnation ratio (% w/w)	Activating temperature (°C)	Activating time (min)	Surface area (m^2^/g)	q_max_ (mg/g)	Adsorbate	Removal efficiency (%)	Reference
**MOSW**^a^	30	700	120	790	61	Cadmium	78	[168]
**Walnut shell**	2	500	90	789	6.1	Zinc	Nil	[169]
**Coconut shell**	Nil	900	120	1344.83	4.31	Phenols	Nil	[170]
**Coffee residue**	100	600	60	1003	89.29	Lead	83.5	[171]
**Foxtail palm fruit**	20	500	120	Nil	2.5	Iron [172]	99.9	[173]
**Rice husk**	2.5	500	120	1365	Nil	Leachate	84	[20]
**Edulis seed**	60	300	180	841	74.31	Cationic dyes	99.59	[174]
**Banana peel**	20	600	120	27.41	27.355	Iron	99.95	[175]
**Coconut coir**	50 (%v/v)	450	60	Nil	41.1	Pb [172]	92.17	[176]
**Vitis vinifera leaf**	3	750	120	295.5	78.9	PFOA^b^	95	[177]
**African palm fruit**	50 (%v/v)	300	120	Nil	Nil	Copper	96.7	[178]

a Municipal solid waste

b Perfluorooctanoic acid

### 3.5 Special features and related application

**Figure 5:**
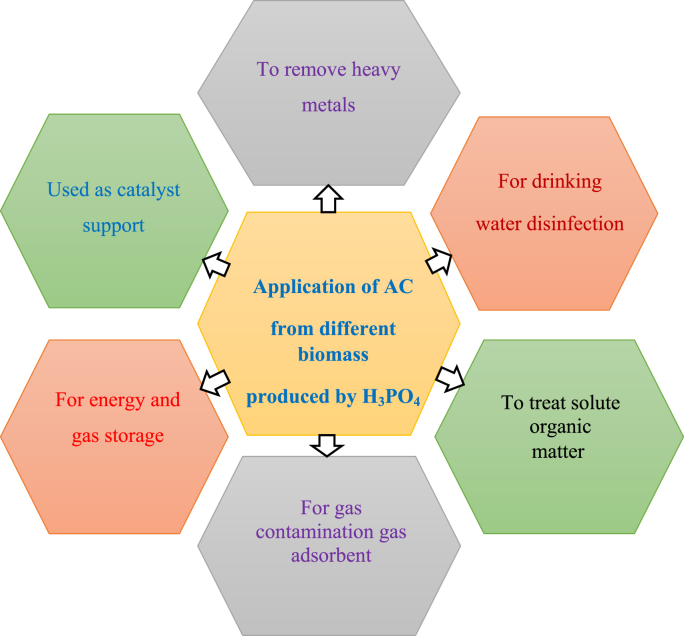
Basic application of activated carbon produced from lignocellulosic biomass via a phosphoric acid agent.

Activated carbon (AC) is a very important adsorbent as a result of its high pore structure, surface area, and high grade of surface reactivity. Furthermore, activated carbon is an economical adsorbent applied for drinking water purification, automotive applications, food-grade products, industrial gas purification, and metal recovery [161]. Hence, this adsorbent (AC) is prepared by modifying the synthesis conditions of the chemical activation process. In a chemical activation process, as discussed in section 1.2, the carbon-containing biomass is mixed with a chemical inhibiting the creation of tar (e.g., sulfuric acid, zinc chloride, potassium hydroxide, phosphoric acid, etc.) and, later, pyrolyzing and washing, the last AC is synthesized. H_3_PO_4_ is preferred over other chemical agents because of corrosion problems, incompetent chemical retrieval, and eco-friendly drawbacks related to other chemicals [162]. Currently, the use of AC procedures has become broadly well-known in drinking water treatment, groundwater restoration, contaminant gas adsorbents [163], metal ion removal [164], catalyst support [165], volatile organic compounds (VOC) [166], and the handling of service water [154]. Similarly, activated carbon is being applied to an increasing amount of wastewater treatment, whether it is in the methodical treatment of specific sewage streams [167]. Figure 5, represented the main application of activated carbon synthesized by phosphoric acid.

#### 3.5.1 Activated carbon for drinking water disinfection

Generally, sedimentation and sand filtration are the most common water treatment techniques. However, these techniques only remove about 20–30% of pollutants. Chlorine-based disinfection techniques proficiently remove microorganisms, but they produce carcinogenic disinfection by-products in the water [118]. Ultraviolet is also another method used to kill microorganisms from the water. However, it is costly and needs power sources to operate [179]. AC is broadly used in drinking water action in countless parts, including the elimination of heavy metals, organic matter, unpleasant taste, and micro-organisms [180]. Especially in recent times, impregnated activated carbon with inorganic nanoparticles (i.e., silver, zinc, copper, gold, and tin oxide) is the most common method of water disinfection due to microorganism growth on the surface of un-impregnated AC [181]. Hence, impregnated activated carbon by inorganic nanoparticles can kill bacteria like E. coli and protozoans like Entamoeba efficiently and simply by drinking water.

#### 3.5.2 Activated carbon for removal of solute organic matter

Natural organic matter in water contains a diversity of compounds like humic acid, fulvic acid, proteins, carbohydrates, and carboxylic acid resulting from the microbial activities and deterioration of plants and animal residues [182]. According to previous studies, activated carbon can be used to remove organic matter based on the physicochemical characteristics (i.e., initial concentration, pH value, molecular size distribution, ionic strength, dissolved oxygen) of natural organic matter from water. The absorbability of organic matter on AC rises with increasing molecular size. This was clarified through the availability of adsorption sites in apertures for slight molecules. Organic matter is negatively charged at neutral pH and electrostatic repulsion or attraction between activated carbon and activated carbon surface charge depends on the amount of acid and basic functional groups [183].

#### 3.5.3 Activated carbon for energy storage

Activated carbon (AC) is used in energy storage as a result of its excellent power density, long life cycle, good pore size, and extensive operating temperature variety [53, 184]. Because of its high conductivity, unique properties, and good electrochemical cycling, activated carbon biomass is in high demand as a conductor in electrochemical capacitances [185, 186]. The electrochemical capacities of ozone-treated carbon make it an especially appealing material for the construction of an electrochemical capacitor [185].

#### 3.5.4 Activated carbon for gas storage

Adsorption of natural gas is a process in which natural gas is deposited on the surface of a porous material at moderately low pressures [186, 187]. The discharge of carbon dioxide (CO_2_) into the air raises the earth's greenhouse outcome, leading to a rise in the worldwide temperature. A probable resolution is an imprisonment and confiscation [188]. For such gas storage, AC is the farthest mutual adsorbent. Some researchers have emphasized the significance of the porosity and the density of the extreme gas storing volume. Both its adsorption capacity and its density have to be exploited [188, 189]. Adsorption kinetics, the heat of adsorption, and adsorption capacity are the main characteristics of activated carbon to store natural gases [190].

#### 3.5.5 Activated carbon for removal of heavy metal

Heavy metals like Chromium, Copper, Zinc, Cobalt, Manganese, Cadmium, Lead, and Nickel that are natural constituents of the Earth's crust are typically related to toxicity [191]. The presence of those metals in the environment has effects on humans if the suitable levels are surpassed [192] and they are also present in ground and surface water [193]. Because of its accessibility, extensive surface area, porosity structure, and good adsorption volume, activated carbon is the furthermost economical, easiest, and most effective adsorbent material for the removal of heavy metals present in the environment (i.e., water and industrial waste). Table 4 shows also the removal percentages of heavy metals, organic matter, and others with phosphoric acid-impregnated activated carbon.

#### 3.5.6 Activated carbon for contaminant gas adsorbents

Fossil fuel burning makes gaseous contaminants, producingdifferences in atmospheric composition. Sulfur dioxide (SO_2_), nitrogen oxides (NO, and particulate matter are themain components of air contamination and are the mostsources of environmental destruction and numerousillnesses, for example, cancer [194]. Gas contaminants primarily damage the immunological hematologic, inhaling ophthalmological, cardiologydermatological neuropsychiatric, and reproductive system [195]. AC obtained from biomass by phosphoric acid, usually prepared as waste, has been studied with a potential catalyst for selective catalytic reduction of nitrogen dioxide [196] and sulfur dioxide [197].

#### 3.5.7 The use of activated carbon as catalyst support

Lots of present-day's chemical processes need the usage of a catalyst supported by a carrier. The higher interior surface area, high inactivity, and flexibility produce activated carbons with ideal support in much utilization including valuable metal catalysis (e.g., Au, Pt, Pd,Ru, Rh, etc.) and base metal catalysts (e.g., Ni, Co, Cu, Zn, Fe) [198, 199]. As catalyst and catalyst support, activated carbon substance display considerable benefit in terms of activity, stabilization, and regenerability on metal or metal oxide catalysts. AC has all the required characteristics to be used as catalyst support andthey have special characteristics for example constancy in either acidic or basic media [165]. AC substantially enhanced the gasification achievement of biomass composition when it was used as catalyst support for platinum metal for the successful transformation of biomass hydrolysis to hydrogen with aqueous-phase revision [198].

## 4 Comparable evidence of AC yield and the surface area produced by H_3_PO_4_ with previous work

The use of phosphoric acidcan synthesize higher biomass-activated carbon yield and surface area as compared to other chemical agents [18]. Phosphoric acid prevents the waste of biomass via vaporization during thermal action, hence rising the activated carbon yield from carbonation, and also chemically bridges to the lignocellulosic biomass polymers and establishes phosphate groups creating cross-links [200]. Table 5 shows a contrast of theyield and surface area of biomass-activated carbon produced by phosphoric acid with other chemical agents stated in the literature.

**Table 5:** Biomass AC yield synthesized by H_3_PO_4_ from different lignocellulosic materials comparison with different chemical agents.

Precursors	Chemical agents	Yield (%)	BET Surface Area (m^2^/g)	References
**Durian shell**	KOH	43.5	979	[115]
	H_3_PO_4_	63	1024	[201]
**Coconut shell**	KOH	32	478	[202]
	H_3_PO_4_	36.9	891	[203]
	ZnCl_2_	50.3	544.7	[204]
**Rice husk**	H_3_PO_4_	44.6	1820	[205]
	ZnCl_2_	32	578	[206]
	H_2_SO_4_	36	681	[17]
	NaOH	---	527	[207]
**Palm shell**	H_3_PO_4_	55	1109	[150]
	ZnCl_2_	38	743	[208]
	KOH	19.3	217	[209]

### 4.1 Prospects and challenges of phosphoric acid impregnated AC

Phosphoric acid was commonly used as a chemical activating agent for helping bond cleavage reactions and protecting the inside hole structure at the same time in chemical activation [210]. Phosphoric acid has two mutual features. It activates the biomass/precursor by entering the pores of the raw materials or biomass. Additionally, it is acidic, which is valuable in breaking chemical bonds and producing an increase in porosity [15]. Nevertheless, few research studies have discussed the challenges of activation by phosphoric acid. Precursor activation and pyrolysis by H_3_PO_4_ take 2–8 h, and phosphate ions can cause interference by precipitating a few elements to be analyzed [211].

## 5 Conclusion and perspectives

The review of lignocellulosic biomass as precursors for the synthesis of AC has been revised based on an important number of appropriate papers available up to now. The chemical activator agent is the main factor governing the efficiency and pertinency of biomass-activated carbon. Now, this paper review; H_3_PO_4_ as a chemical activating agent of lignocellulosic biomass for the synthesis of AC. These lignocellulosic biomasses are in the form of shells, husks, leaves, and grass, and are consequently used as adsorbents. According to the review, the yield, surface area, porosity, and surface functional groups of activated carbon adsorbents highly depend on activated temperature, activated time, and the acid-to precursor ratio of phosphoric acid. The synthesis of activated carbon by phosphoric acid has numerous advantages, like the acceptance of it in food, water, and non-contaminant in nature over other chemical activating agents. Various studies have been conducted on the application of activated carbon produced by phosphoric acid to the removal of organic materials, heavy metals (i.e., lead, copper, uranium, iron, manganese, zinc), and pollutants. The use of activated carbon from numerous lignocellulosic biomasses for pollutant removal is very widespread as a result of its good surface area and good porosity structure. However, there are various inadequacies in the use of modified biomass-activated carbon by inorganic nanoparticles for the elimination of contaminants. Based on the earmark contaminants, lignocellulosic biomass activated carbon impregnated with inorganic nanoparticles for water disinfection may be the best possible, rarely exceeding commercial activated carbons. Hence, with high performance and best impregnated with inorganic nanoparticles as per the elimination of contaminants like pathogens-bacteria from drinking water, the low-cost biomass activated carbon will positively occur in the future disinfectant adsorbent with the good killing of bacteria from contaminated water.

## Declarations

### Author contribution statement

All authors listed have significantly contributed to the development and the writing of this article.

### Funding statement

This work was supported by Addis Ababa Science and Technology University.

### Data availability statement

No data was used for the research described in the article.

### Declaration of interest’s statement

The authors declare no conflict of interest.

### Additional information

No additional information is available for this paper.
